# *Anopheles arabiensis*seasonal densities and infection rates in relation to landscape classes and climatic parameters in a Sahelian area of Senegal

**DOI:** 10.1186/s12879-014-0711-0

**Published:** 2014-12-20

**Authors:** El Hadji Malick Ngom, Ndèye Diango Faye, Cheikh Talla, El Hadji Ndiaye, Jacques-André Ndione, Ousmane Faye, Yamar Ba, Mawlouth Diallo, Ibrahima Dia

**Affiliations:** Unité d’entomologie médicale, Institut Pasteur de Dakar, 36 Avenue Pasteur, Dakar, BP 220 Senegal; Université Cheikh Anta Diop de Dakar, Dakar, Senegal; Université Gaston Berger, Saint-Louis, Senegal; Centre de Suivi Ecologique, Dakar, Senegal

**Keywords:** Anopheles arabiensis, Sahelian area, Landscape classes, Climatic parameters, Malaria transmission

## Abstract

**Background:**

The influence of environmental and climatic factors on malaria vector bionomics and transmission is an important topic in the context of climatic change particularly at macro-geographical level. Sahelian areas could be particularly affected due to heterogeneous features including high inter-annual variability in rainfall and others associated parameters. Therefore, baseline information on the impact of environmental and climatic factors on malaria transmission at micro-geographical level is required for vector risk management and implementation of control strategies.

**Methods:**

Malaria vectors were collected indoors by pyrethrum spray catches in 14 villages belonging to 4 different landscape classes (wooded savanna, shrubby savanna, bare soils and steppe) in the sylvo-pastoral area of Senegal. *Plasmodium falciparum* infection rates were determined using an indirect enzyme-linked immunosorbent assay (ELISA).

**Results:**

*An. arabiensis* was the predominant species in all landscape classes and was the only species collected at the end of the rainy season excepted in villages located in bare soils where it cohabited with *An. coluzzii*. Mean temperature and relative humidity showed similar variations in all the landscape classes covered whereas rainfall was more heterogeneous in terms of pattern, frequency and amount. The mean densities of *An. arabiensis* displayed high seasonal differences with peaks observed in August or September. A positive non-significant correlation was observed between *An. arabiensis* densities for rainfall and humidity whereas a negative non-significant correlation was reported for temperature. *Plasmodium falciparum*-infected mosquitoes were detected only in wooded savanna and bare soils villages.

**Conclusions:**

These observations suggest key roles played by landscape classes and rainfall in malaria vector densities, infection rates and malaria transmission that could be more pronounced in villages situated in wooded savanna and bare soils. Due to the close relationship between environmental and meteorological parameters in this Sahelian region, additional studies on the impact of these parameters are required to further ascertain their association with entomological parameters involved in malaria transmission. From the public health point of view, such information could be useful for human population settlements as well as for monitoring and modelling purposes giving early warning system for implementation of interventions in these unstable transmission zones.

**Electronic supplementary material:**

The online version of this article (doi:10.1186/s12879-014-0711-0) contains supplementary material, which is available to authorized users.

## Background

Many vector-borne diseases occur in tropical and sub-tropical areas where climatic and environmental conditions are favourable for their propagation [1]. The recent observations on climatic changes worldwide are causing panic and their impact on these diseases are still not well established. Many studies suggest that climate change would lead to a resurgence of vector-borne diseases like malaria [2],[3] and it is well known that malaria transmission and prevalence could be highly influenced by spatial and temporal changes in the environment [4]. This is particularly important in non-endemic areas characterised by a low level of malaria transmission and a high variability of climatic and environmental parameters, where a comeback or resurgence of malaria with a high risk of morbidity and mortality in all age groups is expected. For instance, recent studies conducted in western Kenya highlands have suggested an increase of malaria epidemics attributed to changes in land cover [5],[6]. Similar observations are expected in the Sahel (southern edge of the Saharan desert) characterized by heterogeneous features including high inter-annual variability in rainfall [7]. The human populations of this rural area are mainly peasant farmers who depend on seasonal rainfall for agriculture and livestock rearing to generate food and financial gains. On the other hand, water and pastural availability determine human settlements with a direct impact on land cover and use.

While the impact of land use or agricultural practices on malaria vectors occurrence and distribution at macro-geographical levels has been addressed in many situations [8],[9], there is very scarce data at micro-geographical levels. Recent findings indicate that small-scale differences within an area may have important consequences [10],[11] on malaria epidemiology. Krefis et al. [12] showed a direct link of an increase or decrease of malaria risk depending on the types of land cover. For malaria vectors, few studies were carried out on the correlation between environmental parameters such as land cover or use and adults stages of malaria vectors contrary to the aquatic stages [13],[14]. However, as adult vector abundance is positively associated with the availability of aquatic habitats [15],[16], results on land cover changes on aquatic stages are usually extrapolated to estimate adult vector densities.

In the present study, we examined the influence of different landscape classes on malaria vector abundance and infection rates in a Sahelian area of Senegal characterised naturally by a low level of malaria transmission, high inter-annual variability of climate changes [17] and high morbidity rates during seasons with heavy rainfall [18]. This information is important for the understanding of environmental determinants of malaria transmission heterogeneity at a micro-geographical level to assess vector pressure, risk management and implementation of control strategies.

## Methods

### Description of the study area

**Figure 1:**
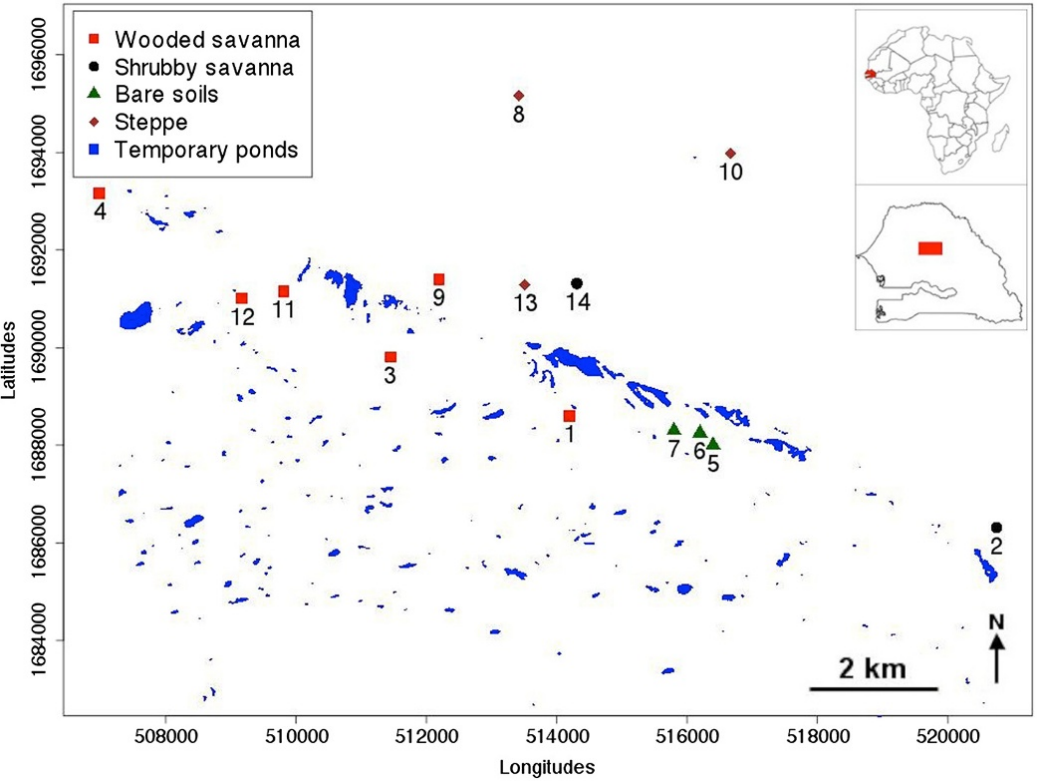
**Localisation of the study sites.** Numbers indicate the sites ID presented in Table 1. Data points are projected in the UTM zone 28 N. Weather stations were installed in Barkedji (ID:1), Keur Alpha (2), Keur Aliou (6) and Keur Diallo (10).

The current study was conducted in 14 villages situated around the village of Barkedji (14°52′04 W-15°16′42 N) in the Sylvo-pastoral area of Senegal (Figure 1) from July to November 2009. The climate is typically Sahelian and is characterized by a semi-arid climate with a summer monsoon (the rainy season) that lasts from July to mid-October. The mean annual temperature is 28.8°C with the lowest value (24.4°C) recorded in January and the highest (35.5°C) in May. The study area is characterized by a complex and dense network of ponds located within the fossil Ferlo riverbed that are filled with water during the rainy season but which dry out during the rest of the year (Figure 1). It is characterized by the predominance of sandy-loam soils on which grows a grass cover dominated by shrubs represented mostly by thorny bushes. The population of this area is estimated at 14,200 inhabitants comprising of Fulani (85%), Wolof (12%), Moors and Serer (3%). Human activities are dominated by livestock rearing (mainly cattle, sheep, goats) and agriculture (mainly millet).

No specific permission was required for work in each of the selected villages. After explaining the purpose of this study, verbal consent was obtained from the village chief as well as from heads of households. Vector control measures used in the study area include personal protections and use of long-lasting insecticidal nets (LLINs) at community level.

### Weather and environmental data collection

Climatic data were collected using weather stations (Campbell scientific BW200) installed near the villages of Barkedji, Keur Alpha, Keur Aliou and Keur Diallo (Figure 1).

The landscape class was chosen as environmental parameter. Landscape classes were defined using remote sensing and geospatial analyses from a SPOT 5 satellite image based on the description of the vegetation classes according to the combination of the FAO and CSA systems [19],[20]. All 14 sampling sites were geo-referenced with a hand-held GPS receiver and each of them was classified to the corresponding landscape class. Four landscape classes were identified in the study area. Table 1 and Figure 1 present each of the 14 villages and the corresponding landscape type.

**Table 1:** **Main characteristics of studied villages**

Sites ID	Villages	Longitudes	Latitudes	Landscape classes
1	Barkedji	15°17′0″N	14°53′0″W	Wooded savanna
2	Keur Alpha	15°14′42″N	14°47′57″W	Shrubby savanna
3	Dague Nabe	15°17′04″N	14°52′96″W	Wooded savanna
4	Diabal	15°18′53″N	14°56′05″W	Wooded savanna
5	Keur Dadal	15°16′23″N	14°51′6″W	Bare soils
6	Keur Aliou	15°15′86″N	14°50′30″W	Bare soils
7	Keur Gallo	15°16′25″N	14°51′01″W	Bare soils
8	Keur Bandji	15°19′34″N	14°52′20″W	Steppe
9	Keur Bathiel	15°17′32″N	14°52′43″W	Wooded savanna
10	Keur Diallo	15°19′10″N	14°50′24″W	Steppe
11	Niakha	15°17′28″N	15°54′18″W	Wooded savanna
12	Niakha Ndiaybe	15°15′56″N	14°50′18″W	Wooded savanna
13	Keur Daha	15°17′31″N	14°52′17″W	Steppe
14	Wouro Thilli	15°17′31″N	14°51′36″W	Shrubby savanna

### Mosquitoes sampling and processing

Daytime indoor resting mosquitoes were collected monthly within the same selected houses from all 14 villages by pyrethrum spray catches (PSC). Upon collection, *Anopheles* females were sorted and identified to species using the morphological key of Gillies and De Meillon [21]. For the known malaria vectors already described in the study area, the physiological status was recorded, then all mosquitoes were stored individually in labelled vials containing silica gel for subsequent analyses. In the laboratory, the mosquitoes from the *Anopheles gambiae* complex were identified using the molecular method of Fanello et al. [22]. The heads and thoraces of all anopheline females were tested by ELISA for the detection of *Plasmodium falciparum* circumsporozoite protein (CSP) using the procedure of Wirtz et al. [23].

### Data analysis

To study the association between landscape classes and entomological parameters (densities, infection rates), each of the 14 villages was classified to the corresponding landscape class. For analysis of the distribution between landscape classes’, monthly anopheline females’ densities were calculated as the number of specimens per room (SPR) and log transformed to normalize the distribution. The Shapiro-Wilk test was used to test the normality of the data and Levene’s test for equality of variances. Subsequently, the differences between landscape classes, mean densities and collection months were analysed using analysis of variance (ANOVA) followed by Tukey-Kramer post-hoc tests. The circum-sporozoite protein infection rates (CSP-IR) was calculated as the proportion of females found to contain the CS protein. To study the associations between the temporal distribution of the vector densities and rainfall, mean relative humidity and temperature, the data from four villages representative of the 4 landcape classes were pooled and the associations evaluated by the Pearson correlation. All these analyses were performed using the R software (version 2.14.1).

## Results

### Mosquito collection

A total of 7652 *Anopheles* specimens belonging to 6 species were collected by PSC from the four landscape classes (Table 2). *An. gambiae* s.l. was the predominant species in all landscape classes. *An. rufipes* was also collected in all landscape classes but was more frequent in villages located in wooded and shrubby savanna. *An. funestus*, *An. pharoensis* and *An. domicola* were collected only in wooded savanna villages whereas *An. welcomei* was scarce and observed in bare soils villages.

**Table 2:** **Number and abundance (%) of anopheline species collected in each of the four landscape classes**

Landscape classes/	***An. funestus***		***An. gambiae***		***An. pharoensis***		***An. rufipes***		***An. welcomei***		***An. domicola***	
villages	N	%	N	%	N	%	N	%	N	%	N	%
Wooded savanna												
Barkedji	4	0.10	3670	90.82	5	0.12	362	8.96	0	0.00	0	0.00
Dague Nabe	1	0.24	404	95.28	0	0.00	18	4.25	0	0.00	1	0.24
Diabal	1	0.13	681	91.53	0	0.00	62	8.33	0	0.00	0	0.00
Keur Bathiel	0	0.00	42	85.71	0	0.00	7	14.29	0	0.00	0	0.00
Niakha	1	0.12	717	82.99	1	0.12	145	16.78	0	0.00	0	0.00
Niakha Ndiaybe	1	0.14	602	83.15	0	0.00	121	16.71	0	0.00	0	0.00
Shrubby savanna												
Keur Alpha	0	0.00	139	96.53	0	0.00	5	3.47	0	0.00	0	0.00
Wouro Thilli	0	0.00	28	90.32	0	0.00	3	9.68	0	0.00	0	0.00
Bare soils												
Keur Dadal	0	0.00	231	94.67	0	0.00	13	5.33	0	0.00	0	0.00
Keur Aliou	0	0.00	55	93.22	0	0.00	4	6.78	0	0.00	0	0.00
Keur Gallo	0	0.00	85	93.41	0	0.00	5	5.49	1	1.10	0	0.00
Steppe												
Keur Bandji	0	0.00	142	98.61	0	0.00	2	1.39	0	0.00	0	0.00
Keur Daha	0	0.00	46	97.87	0	0.00	1	2.13	0	0.00	0	0.00
Keur Diallo	0	0.00	44	95.65	0	0.00	2	4.35	0	0.00	0	0.00
Total	8	0.10	6886	89.99	6	0.08	750	9.80	1	0.01	1	0.01

N = number, % = percentage.

### Distribution pattern of *An. gambiae*complex within the study area

Overall, 863 specimens belonging to the *An. gambiae* complex were molecularly identified. Only *An. arabiensis* and *An. coluzzii* were present in the study area; *An. arabiensis* was the most predominant species with 81.8% (706/863) in the collection. No statistically significant difference was observed in the proportions of *An. arabiensis* in the different landscape classes (*χ*^2^ = 4.45, df = 3, p = 0.22). The relative proportions of the two species fluctuated based on the landscape class and month of collection.

*An. arabiensis* was the most abundant species in wooded and shrubby savanna villages at any time point during the months of collections and moreover, it was the only species collected at the end of the rainy season in November (Table 3 and Additional file 1). *An. coluzzii* on the other hand was absent or had very low proportions at the beginning of the rainy season in July (see Additional file 2). Its abundances increased gradually reaching maximum levels in August or September before it started to decline steadily. In shrubby savanna villages *An. coluzzii* was collected only in August and September with the respective frequencies of 42.9% and 11.1% (Table 3 and Additional file 2).

**Table 3:** **Monthly variations of** ***An. arabiensis*** **mean proportions (±se) in each of the four landscape classes**

Landscape classes	Study period				
	July	August	September	October	November
Wooded savanna	89.9 ± 3.4^b^	59 ± 12.1^a^	88.8 ± 5.1^b^	98 ± 2^b^	100^b^
Shrubby savanna	100^c^	57.1^a^	88.7 ± 1.3^b^	100^c^	100^c^
Bare soils	-	72.7 ± 13.8^a^	92.5 ± 3.8^a^	93.3 ± 6.7^a^	90 ± 10^a^
Steppe	75 ± 25^a,b^	65.3 ± 1.4^a,b^	53.3 ± 13.3^a^	66.7 ± 33.3^a^	100^b^

For the different landscape classes, means with different letters are significantly different (p < 0.05).

Comparisons were made between months for each of the four landscape classes. Standard errors not presented are null.

In bare soils and steppe landscapes, the two species were collected almost throughout the whole collection period (4 out of the 5 months collection period). The lowest frequencies of *An. arabiensis* were observed in August and September respectively (Table 3 and Additional file 2).

### Monthly densities of *Anopheles gambiae*mosquitoes

**Figure 2:**
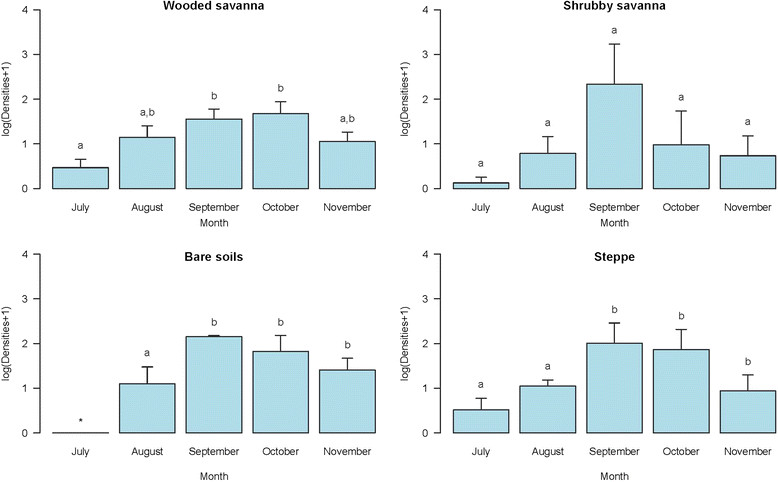
**Temporal variations of the mean densities of** ***An. arabiensis*** **in each of the four landscape classes.** For the different landscape classes, means with different letters are significantly different (p < 0.05). *no mosquito collected.

Overall, 2695 rooms were sprayed during the surveys carried out resulting in the collection of 6886 *An. gambiae* mosquitoes. Overall, the mean number of specimens per room (SPR) was estimated to be 3.5. The means for the villages situated in the different landscapes (wooded savanna, shrubby savanna, bare soils and steppe) were 3 ± 0.45, 3.79 ± 2.32, 3.87 ± 0.84, 3.94 ± 1.25 respectively. These means were statistically comparable (F = 0.26, p = 0.85). However, statistically significant differences were observed between the monthly means in wooded savanna and bare soils villages (Figure 2). Overall, the mean densities were low at the beginning of the surveys in July (beginning of rainy season) with a maximum of 1 SPR in all landscape classes. They increased steadily thereafter reaching a peak in September for shrubby savanna villages, bare soils and steppe whilst for wooded savanna villages the peak was observed in October (Figure 2).

**Figure 3:**
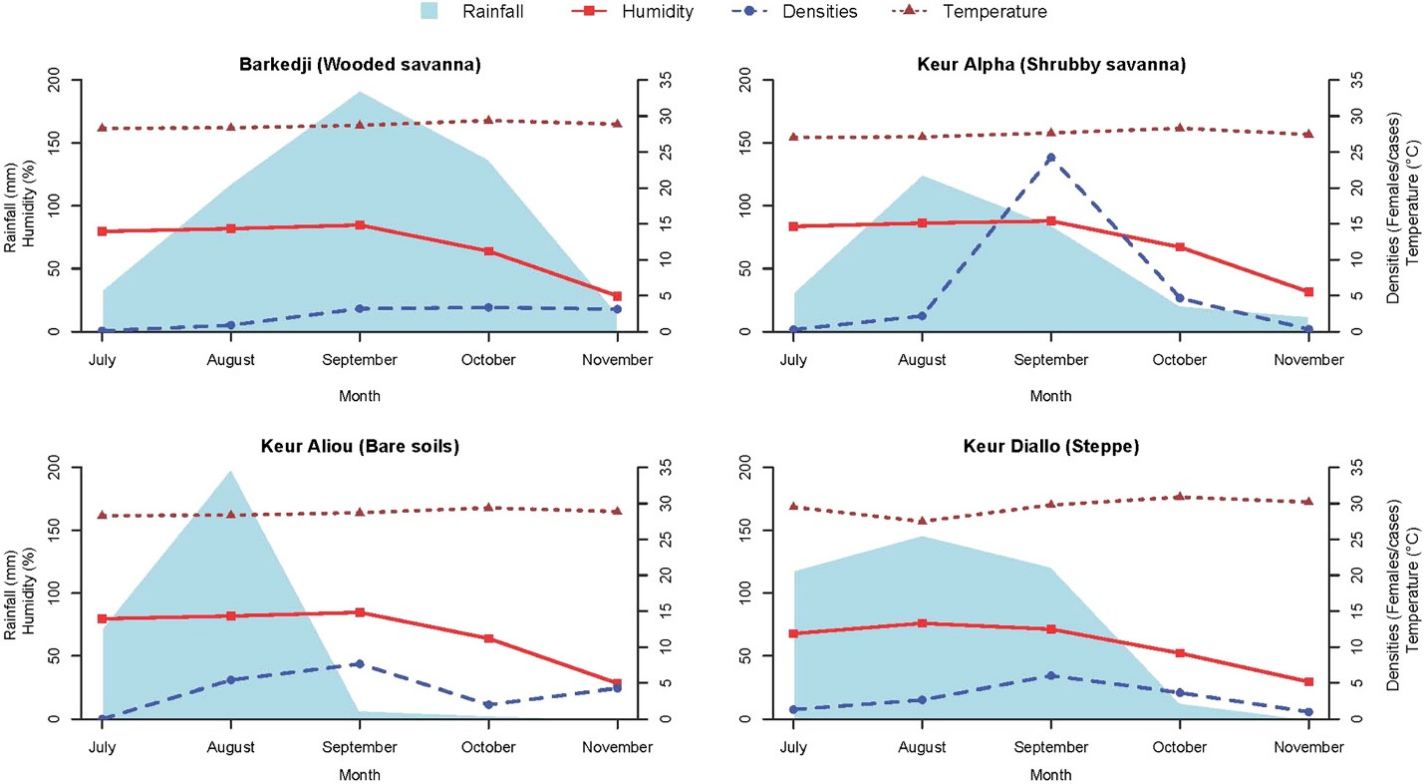
**Variations of** ***An. arabiensis*** **densities in relation to rainfall, temperature and humidity in each of four villages representative of the four landscape classes.**

### Influence of climate parameters on *Anopheles*densities

This influence was studied in 4 sites representing each of the four landscape classes: Barkedji (wooded savanna), Keur Alpha (shrubby savanna), Keur Aliou (Bare soils) and Keur Diallo (Steppe). In each of these sites, the mean temperature and mean relative humidity showed similar variations whereas the rainfall was heterogeneous in terms of patterns, frequency and quantity. Rainfall peaked in September in Barkedji village and in the three other villages it peaked in August (Figure 3). The highest number of SPR was observed soon after the rainfall peaked either in October and September in Barkedji or September and August for the three other villages. A positive but not significant correlation was observed with rainfall (Pearson r = 0.09, p = 0.71) and humidity (Pearson r = 0.25, p = 0.29) while a negative non-significant correlation was observed for temperature (Pearson r = −0.09, p = 0.68) (see Additional file 3).

### Circumsporozoite infection rates

The head and thoraces of 3768 *An. gambiae* s.l. females were tested by ELISA for *Plasmodium falciparum* CS antigen detection (Table 4). No specimen collected from villages situated in shrubby savanna and steppe were CS positive. Overall 11 positive females were detected, 10 in villages situated in wooded savanna (6 collected in Barkedji in August, 2 in Niakha in September and 2 in Niakha Ndiaybe in September) and 1 in a village within bare soils (Keur Aliou in August). The mean infection rates were 0.34% in wooded savanna and 0.37% in bare soils, respectively. No statistically significant difference was observed between the four landscape classes (*χ*^2^ = 0.84, df = 3, p = 0.84). A similar result was observed between wooded savanna villages (*χ*^2^ = 2.49, df = 5, p = 0.78) whereas the infection rates were significantly different between bare soils villages (*χ*^2^ = 13.94, df = 2, p < 0.001).

**Table 4:** ***An. arabiensis*** **mean infection rates calculated by enzyme-linked immunosorbent assay for** ***P. falciparum*** **in the different villages/lansdcape classes**

Villages	Negative	Positive	Total	CSP-IR (%)	95% CI
Wooded savanna					
Barkedji	1705	6	1711	0.35	0.16-0.76
Dague Nabe	227	0	227	0	-
Diabal	301	0	301	0	-
Keur Bathiel	29	0	29	0	-
Niakha	606	2	608	0.33	0.09-1.19
Niakha Ndiaybe	367	2	369	0.54	0.15-1.95
Shrubby savanna					
Keur Alpha	110	0	110	0	-
Wouro Thilli	18	0	18	0	-
Bare soils					
Keur Dadal	195	0	195	0	-
Keur Aliou	17	1	18	5.55	0.28-25.76
Keur Gallo	55	0	55	0	-
Steppe					
Keur Bandji	80	0	80	0	-
Keur Daha	17	0	17	0	-
Keur Diallo	30	0	30	0	-
Total	3757	11	3768	0.29	0.16-0.52

CSP-IR = Circumsporozoite protein infection rate, CI = confidence interval based on a binomial distribution.

## Discussion

All the anopheline species collected during this study were already described in the area [24]. However, the list is not exhaustive since other known species in the area were not collected even though the study was conducted longitudinally. This is due primarily to the collection method used that may have targeted endophilic species closely associated with human environments.

*An. arabiensis* was the most common species during this study. *An. pharoensis* and *An. funestus,* also vectors of malaria in Senegal were present but at very low proportions. *An. pharoensis* was previously described in the area [25] whereas *An. funestus* was never observed during previous malaria vector studies conducted in the area [17],[26]. The presence of *An. funestus* therefore confirms the comeback of this species in the Senegal River basin which was described in a recent study [27]. This species was first described in the Senegal River basin in the 1970s, but thereafter it disappeared following recurrent droughts only to appear again in 1999 in the low valley of the Senegal River [28]. Its presence in the current study area extends the re-colonization zone to the Ferlo area. This is perhaps due to the restoration and presence of its breeding sites that consist of natural/artificial permanent and semi-permanent water bodies with floating or emerging vegetation. This study area has characteristic favourable breeding sites of this nature constituting temporary pools covered with vegetation emerging from the middle to the end of the rainy season [29].

The presence of *An. funestus* was limited to wooded savanna villages only and at low proportions (0.01%). Therefore, its involvement in malaria transmission is presumed to be low. Like *An. arabiensis*, *An. rufipes* was also observed in all landscape types. Due to the relatively high proportions observed for *An. rufipes*, the determination of the role of this species in malaria transmission needs further investigation to incriminate it, as a recent study conducted in Burkina Faso has demonstrated its involvement in the transmission of human *Plasmodium* [30].

The variations observed in the proportions of the species of the *An. gambiae* complex in the four different landscape classes, could express different adaptive characteristics of these species. *An. arabiensis* was found to be the prevalent species in the whole study area. This observation is in agreement with the distribution of this species, which is located in dry to humid savannah areas [31]. Moreover, it is noteworthy that the highest proportions of this species were observed during the less humid months namely in July and November.

Despite the comparable mean densities observed in the four landscape classes, significant variations were observed when considering the collection months in villages located in wooded savanna and bare soils. Malaria transmission could be higher in these villages during September and October because the highest densities and infected mosquitoes with *P. falciparum* were observed in this period. Additional evidence comes from a recent study conducted in the study area on the spatio-temporal analysis of feeding behaviour [32], which showed that it was mainly during the rainy season that the blood meals taken from human were widespread and homogenously distributed in the study area. The anthropophilic rates were higher in September in villages situated in wooded savanna whereas in bare soils villages, the differences were less marked but the blood meals taken from humans were more uniformly distributed in September. The results of Lemasson et al. [17] is also an evidence to indicate maximal transmission during this period, as they showed that during two successive years, malaria transmission peaks were observed either in October or at the end of September. In shrubby savanna and steppe villages, the absence of infection as well as the seasonal homogeneous variations in densities could lead to a lower transmission in these villages in comparison to wooded savanna and bare soils villages. However, due to the relatively low number of villages involved (small sample size), these results should be confirmed by more intensive and longitudinal studies.

The study of the influence of climatic parameters on densities showed a non-significant positive correlation of rainfall regardless of the landscape class type. This observation is consistent with that of Bi et al. [33]. Indeed, *An. arabiensis* aquatic stages prefer to breed in small, shallow, temporary rain pools or stagnant bodies of water fully exposed to the sun [34]. In our study area, these types of breeding sites are predominant and rainfall-dependent as also observed elsewhere [35],[36]. A positive but not significant correlation was also observed for humidity while a negative non-significant correlation was observed for temperature. These observations could be due to the relative uniformity of humidity and temperature in the 4 landscape classes. This is further sustained by the fact that, if we consider that water temperature influences the development of aquatic stages of *Anopheles* species [37], the uniformity of the temperature contributes to similar influences in the different landscape classes.

## Conclusions

Thus, as environmental conditions are closely related to meteorological data, additional studies with a close follow-up of climatic parameters are needed to ascertain their relationship with entomological parameters. This is reinforced by the fact that climatic parameters can dramatically shift change in mosquito abundance, longevity and infection [38]. From the public health point of view, such information could be useful for human population settlements as well as for monitoring and modelling purposes generating early warning system for implementing interventions.

## Additional files

## Electronic supplementary material

Additional file 1: **Variations of the proportions of** ***An. arabiensis*** **between villages belonging to the same landscape class.** For the different villages belonging to the same landscape class, means with different letters are significantly different (p < 0.05). (PDF 53 KB)

Additional file 2: **Monthly variations of** ***An. arabiensis*** **and** ***An. coluzzii*** **mean proportions in each of the four landscape classes.** (PDF 91 KB)

Additional file 3: **Correlation between** ***An. arabiensis*** **densities and climatic parameters.** (PDF 106 KB)

Below are the links to the authors’ original submitted files for images.

Authors’ original file for figure 1

Authors’ original file for figure 2

Authors’ original file for figure 3

## References

[1] Githeko AK, Lindsay SW, Confalonieri UE, Patz JA (2000). Changement climatique et maladies à transmission vectorielle: une analyse régionale. Bull OMS.

[2] Bouma MJ, Sondorp HE, Van der Kaay HJ (1994). Health and climate change. Lancet.

[3] Colwell RR, Epstein PR, Gubler D, Maynard N, McMichael AJ, Patz JA, Sack RB, Shope R (1998). Climate change and human health. Science.

[4] Rogers DJ, Randolph SE, Snow RW, Hay SI (2002). Satellite imagery in the study and forecast of malaria. Nature.

[5] Lindblade KA, Walker ED, Onapa AW, Katungu J, Wilson ML (2000). Land use change alters malaria transmission parameters by modifying temperature in a highland area of Uganda. Trop Med Int Health.

[6] Patz JA, Graczyk TK, Geller N, Vittor AY (2000). Effects of environmental change on emerging parasitic diseases. Int J Parasitol.

[7] Lebel T, Diedhiou A, Laurent H (2003). Seasonal cycle and interannual variability of the Sahelian rainfall at hydrological scales. J Geophys Res.

[8] Ijumba JN, Shenton FC, Clarke SE, Mosha FW, Lindsay SW (2002). Irrigated crop production is associated with less malaria than traditional agricultural practices in Tanzania. Trans R Soc Trop Med Hyg.

[9] Keiser J, de Castro MC, Maltese MF, Bos R, Tanner M, Singer BH, Utzinger J (2005). Effect of irrigation and large dams on the burden of malaria on a global and regional scale. Am J Trop Med Hyg.

[10] Mwangangi JM, Shililu J, Muturi EJ, Muriu S, Jacob B, Kabiru EW, Mbogo CM, Githure J, Novak RJ (2010). Anopheles larval abundance and diversity in three rice agro-village complexes Mwea irrigation scheme, central Kenya. Malar J.

[11] Jacob BG, Muturi E, Halbig P, Mwangangi J, Wanjogu RK, Mpanga E, Funes J, Shililu J, Githure J, Regens JL, Novak RJ (2007). Environmental abundance of Anopheles (Diptera: Culicidae) larval habitats on land cover change sites in Karima Village, Mwea Rice Scheme, Kenya. Am J Trop Med Hyg.

[12] Krefis AC, Schwarz NG, Nkrumah B, Acquah S, Loag W, Oldeland J, Sarpong N, Adu-Sarkodie Y, Ranft U, May J (2011). Spatial analysis of land cover determinants of malaria incidence in the Ashanti Region. Ghana PLoS One.

[13] Minakawa N, Sonye G, Mogi M, Yan G (2004). Habitat characteristics of *Anopheles gambiae s.s.*larvae in a Kenyan highland. Med Vet Entomol.

[14] Minakawa N, Munga S, Atieli F, Mushinzimana E, Zhou G, Githeko AK, Yan G (2005). Spatial distribution of anopheline larval habitats in western kenyan highlands: effects of land cover types and topography. Am J Trop Med Hyg.

[15] Fillinger U, Sonye G, Killeen GF, Knols BG, Becker N (2004). The practical importance of permanent and semipermanent habitats for controlling aquatic stages of *Anopheles gambiae*sensu lato mosquitoes: operational observations from a rural town in western Kenya. Trop Med Int Health.

[16] Shililu J, Mbogo C, Ghebremeskel T, Githure J, Novak R (2007). Mosquito larval habitats in a semiarid ecosystem in Eritrea: impact of larval habitat management on *Anopheles arabiensis*population. Am J Trop Med Hyg.

[17] Lemasson JJ, Fontenille D, Lochouarn L, Dia I, Simard F, Ba K, Diop A, Diatta M, Molez JF (1997). Comparison of bahavior and vector competence of *A. gambiae* and *A. arabiensis*(Diptera: Culicidae) in Barkedji, a sahelian area of Senegal. J Med Entomol.

[18] Molez JF, Diop A, Gaye O, Lemasson JJ, Fontenille D (2006). Morbidité palustre à Barkedji, village du Ferlo, en zone sahélienne du Sénégal. Bull Soc Path Ex.

[19] FAO: *Africover, classification de l’occupation du sol*. RSC Series; 1997. 70, 80 pp.

[20] Conseil Scientifique pour l’Afrique (CSA) (1956). Phytogéographie (Yangambi).

[21] Gillies MT, De Meillon B (1968). The Anophelinae of Africa South of the Sahara, 2nd edition. Publ South Afr Ins Med Res.

[22] Fanello C, Santolamazza F, Della Torre A (2002). Simultaneous identification of species and molecular forms of the *Anopheles gambiae*complex by PCR-RFLP. Med Vet Entomol.

[23] Wirtz RA, Zavala F, Charoenvit Y, Campbell GH, Burkot TR, Schneider I, Esser KM, Beaudoin RL, Andre RG (1987). Comparative testing of monoclonal antibodies against *Plasmodium falciparum*sporozoites for ELISA development. Bull WHO.

[24] Traoré-Lamizana M, Fontenille D, Diallo M, Bâ Y, Zeller HG, Mondo M, Adam F, Thonon J, Maïga A (2001). Arbovirus surveillance from 1990 to 1995 in the Barkedji area (Ferlo) of Senegal, a possible natural focus of Rift Valley fever virus. J Med Entomol.

[25] Traore-Lamizana M, Zeller HG, Mondo M, Hervy JP, Adam F, Digoutte JP (1994). Isolations of West Nile and Bagaza viruses from mosquitoes (Diptera: Culicidae) in central Senegal (Ferlo). J Med Entomol.

[26] Dia I, Diallo D, Duchemin JB, Ba Y, Konate L, Costantini C, Diallo M (2005). Comparisons of human-landing catches and odor-baited entry traps for sampling malaria vectors in Senegal. J Med Entomol.

[27] Dia I, Konate L, Samb B, Sarr JB, Diop A, Rogerie F, Faye M, Riveau G, Remoue F, Diallo M, Fontenille D (2008). Bionomics of malaria vectors and relationship with malaria transmission and epidemiology in three physiographic zones in the Senegal River Basin. Acta Trop.

[28] Konate L, Diop A, Sy N, Faye MN, Deng Y, Izri A, Faye O, Mouchet J (2001). Comeback of *A. funestus*in Sahelian Senegal. Lancet.

[29] Lacaux JP, Tourre YM, Vignolles C, Ndione JA, Lafaye M (2007). Classification of ponds from high-spatial resolution remote sensing: application to Rift Valley Fever epidemics in Senegal. J R Sensing Env.

[30] Da DF, Diabate A, Mouline K, Lefèvre T, Awono-Ambene HP, Ouedraogo JB, Dabiré KR (2013). Anopheles rufipes remains a potential malaria vector after the first detection of infected specimens in 1960 in Burkina Faso. J Infect Dis Ther.

[31] Coetzee M, Craig M, le Sueur D (2000). Distribution of African malaria mosquitoes belonging to the *Anopheles gambiae*complex. Parasitol Today.

[32] el Ngom HM, Ndione JA, Ba Y, Konaté L, Faye O, Diallo M, Dia I (2013). Spatio-temporal analysis of host preferences and feeding patterns of malaria vectors in the sylvo-pastoral area of Senegal: impact of landscape classes. Parasit Vectors.

[33] Bi P, Tong S, Donald K, Parton K, Ni J (2003). Climatic variables and transmission of malaria: a 12-year data analysis in Shuchen County. Chin Publ Health Rep.

[34] Gimnig JE, Ombok M, Kamau L, Hawley WA (2001). Characteristics of larval anopheline (Diptera: Culicidae) habitats in Western Kenya. J Med Entomol.

[35] Lindblade KA, Walker ED, Onapa AW, Katungu J, Wilson ML (1999). Highland malaria in Uganda: prospective analysis of an epidemic associated with El Niño. Trans R Soc Trop Med Hyg.

[36] Koenraadt CJM, Githeko AK, Takken W (2004). The effects of rainfall and evapotranspiration on the temporal dynamics of *Anopheles gambiae* s.s. and *Anopheles arabiensis*in a Kenyan village. Acta Trop.

[37] Lyimo EO, Takken W, Koella JC (1992). Effect of rearing temperature and larval density on larval survival, age at pupation and adult size of *Anopheles gambiae*. Entomol Exp Appl.

[38] Bomblies A, Eltahir EA (2009). Assessment of the impact of climate shifts on malaria transmission in the Sahel. Ecohealth.

