# Supplementary material for: Anopheles arabiensisseasonal densities and infection rates in relation to landscape classes and climatic parameters in a Sahelian area of Senegal
Source: BMC Infect Dis. 2014 Dec 20;14:3838. doi: 10.1186/s12879-014-0711-0 (PMC4279681; doi:10.1186/s12879-014-0711-0)
Supplement: Supplementary file 1 — Additional file 1: Variations of the proportions of An. arabiensis between villages belonging to the same landscape class. For the different villages belonging to the same landscape class, means with different letters are significantly different (p < 0.05). (PDF 53 KB) [file 12879_2014_711_MOESM1_ESM.pdf]

Additional file 1

Variations of the mean proportions of *An. arabiensis* between villages belonging to the same landscape class. For the different villages belonging to the same landscape class, means with different letters are significantly different ($p<0.05$)

Landscape Classes	Villages	Mean proportions (±se)
Wooded savanna	Barkedji	79.75±3.92[a]
	Dague Nabe	86.07±11.07[a]
	Diabal	95±5[a]
	Keur Bathiel	100[a]
	Niakha	70.83±17.5[a]
	Niakha Ndiaybe	67.96±14.54[a]
Shrubby savanna	Keur Alpha	88.9±8.33[a]
	Wouro Thilli	95±5[a]
Bare soils	Keur Aliou	96.88±3.12[a]
	Keur Dadal	80.62±8.68[a]
	Keur Gallo	83.89±10.56[a]
Steppe	Keur Bandji	74.67±8.98[a]
	Keur Daha	88.89±11.11[a]
	Keur Diallo	80.5±12.46[a]
