# Supplementary material for: Anopheles arabiensisseasonal densities and infection rates in relation to landscape classes and climatic parameters in a Sahelian area of Senegal
Source: BMC Infect Dis. 2014 Dec 20;14:3838. doi: 10.1186/s12879-014-0711-0 (PMC4279681; doi:10.1186/s12879-014-0711-0)

**Additional file 2** : Monthly variations of *An. arabiensis* and *An. coluzzii* mean proportions (±se) in each of the four landscape classes

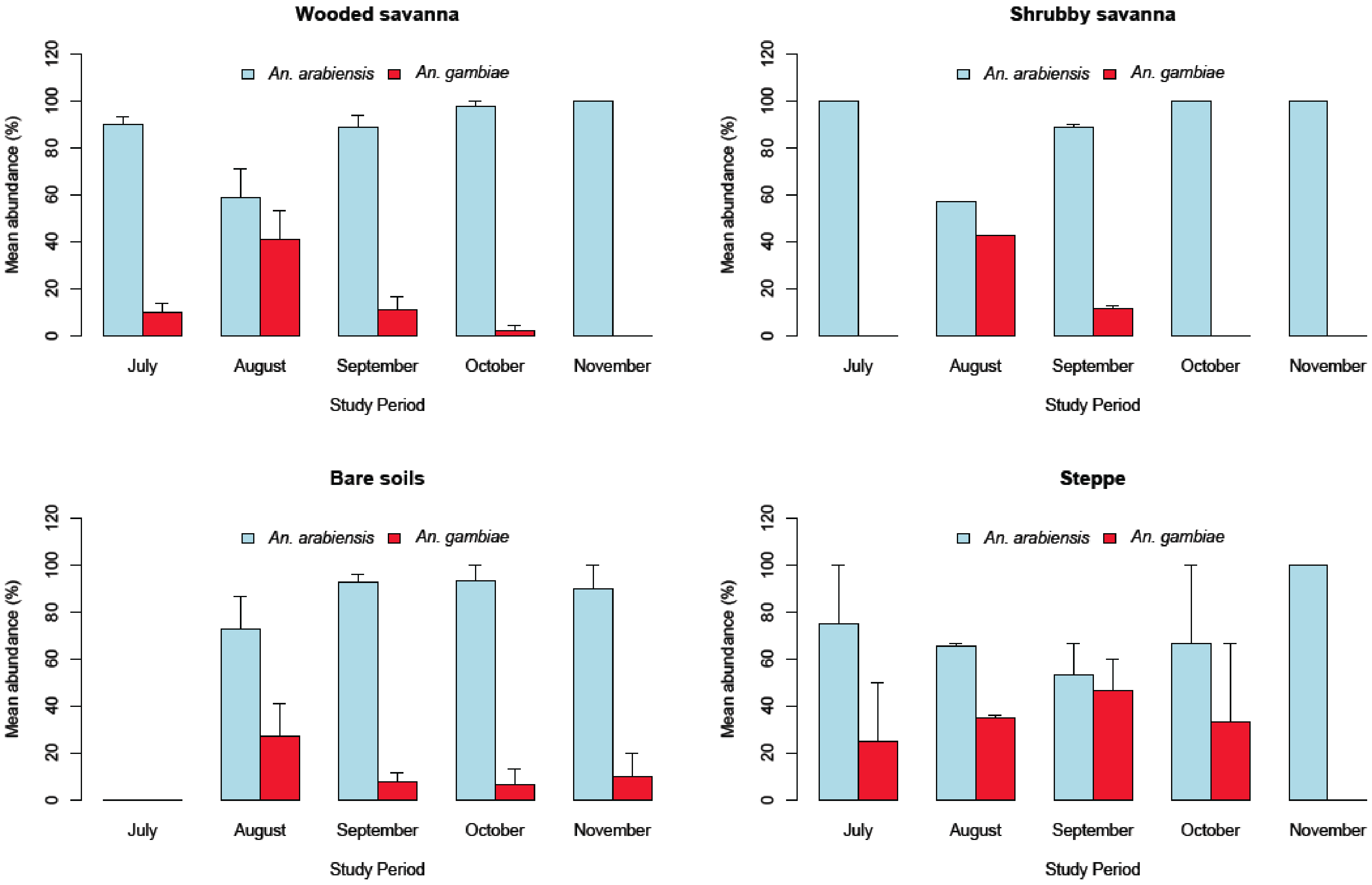

Supplement: Supplementary file 2 — Additional file 2: Monthly variations of An. arabiensis and An. coluzzii mean proportions in each of the four landscape classes. (PDF 91 KB) [file 12879_2014_711_MOESM2_ESM.pdf]
