# Supplementary figures and images for: Anopheles arabiensisseasonal densities and infection rates in relation to landscape classes and climatic parameters in a Sahelian area of Senegal
Source: BMC Infect Dis. 2014 Dec 20;14:3838. doi: 10.1186/s12879-014-0711-0 (PMC4279681; doi:10.1186/s12879-014-0711-0)

## Additional file 3

Correlation between *An. arabiensis* densities and climatic parameters

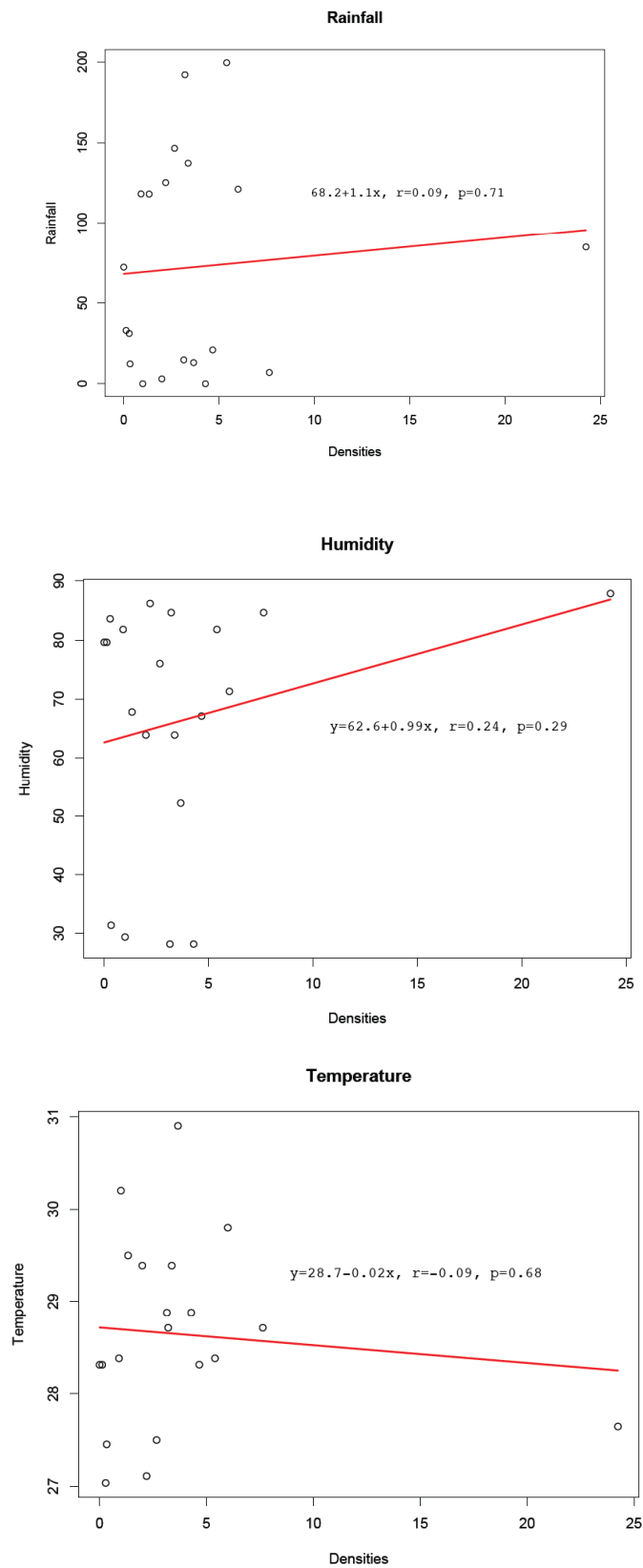

Supplement: Supplementary file 3 — Additional file 3: Correlation between An. arabiensis densities and climatic parameters. (PDF 106 KB) [file 12879_2014_711_MOESM3_ESM.pdf]

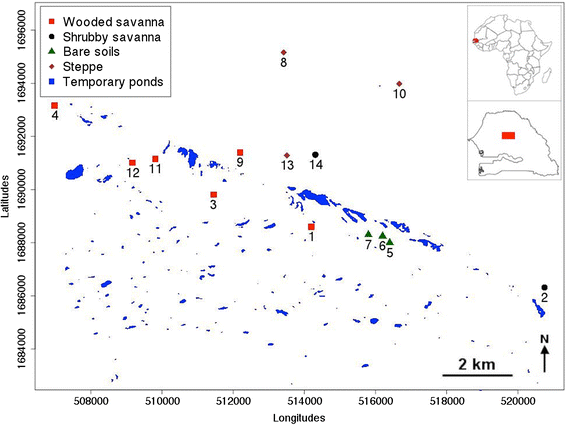

Supplement: Supplementary file 4 — Authors’ original file for figure 1 [file 12879_2014_711_MOESM4_ESM.gif]

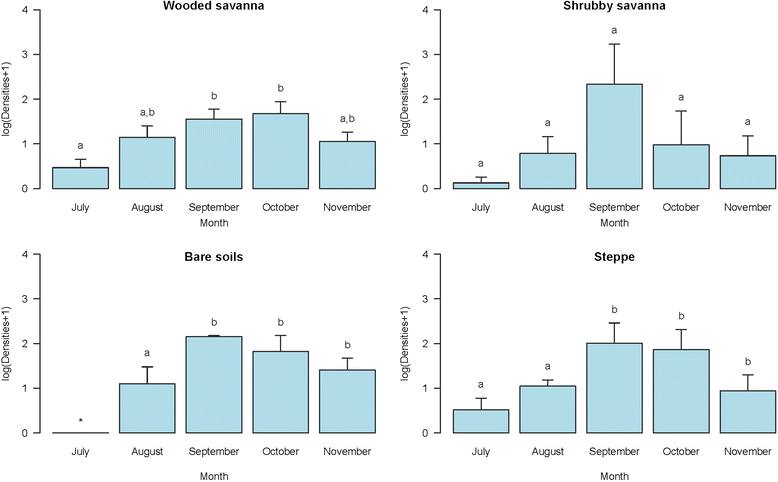

Supplement: Supplementary file 5 — Authors’ original file for figure 2 [file 12879_2014_711_MOESM5_ESM.gif]

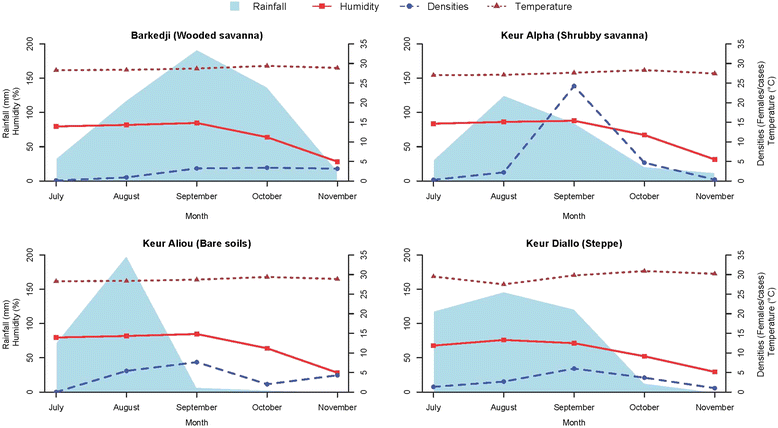

Supplement: Supplementary file 6 — Authors’ original file for figure 3 [file 12879_2014_711_MOESM6_ESM.gif]
